# ISG20L1 is a p53 family target gene that modulates genotoxic stress-induced autophagy

**DOI:** 10.1186/1476-4598-9-95

**Published:** 2010-04-29

**Authors:** Kathryn G Eby, Jennifer M Rosenbluth, Deborah J Mays, Clayton B Marshall, Christopher E Barton, Seema Sinha, Kimberly N Johnson, Luojia Tang, Jennifer A Pietenpol

**Affiliations:** 1 Department of Biochemistry, Center in Molecular Toxicology, Vanderbilt-Ingram Cancer Center, Vanderbilt University School of Medicine, Nashville, TN, USA 37232

## Abstract

**Background:**

Autophagy is characterized by the sequestration of cytoplasm and organelles into multimembrane vesicles and subsequent degradation by the cell's lysosomal system. It is linked to many physiological functions in human cells including stress response, protein degradation, organelle turnover, caspase-independent cell death and tumor suppression. Malignant transformation is frequently associated with deregulation of autophagy and several tumor suppressors can modulate autophagic processes. The tumor suppressor p53 can induce autophagy after metabolic or genotoxic stress through transcriptionally-dependent and -independent mechanisms. In this study we expand on the former mechanism by functionally characterizing a p53 family target gene, ISG20L1 under conditions of genotoxic stress.

**Results:**

We identified a p53 target gene, ISG20L1, and show that transcription of the gene can be regulated by all three p53 family members (p53, p63, and p73). We generated an antibody to ISG20L1 and found that it localizes to the nucleolar and perinucleolar regions of the nucleus and its protein levels increase in a p53- and p73-dependent manner after various forms of genotoxic stress. When ectopically expressed in epithelial cancer-derived cell lines, ISG20L1 expression decreased clonogenic survival without a concomitant elevation in apoptosis and this effect was partially rescued in cells that were ATG5 deficient. Knockdown of ISG20L1 did not alter 5-FU induced apoptosis as assessed by PARP and caspase-3 cleavage, sub-G_1 _content, and DNA laddering. Thus, we investigated the role of ISG20L1 in autophagy, a process commonly associated with type II cell death, and found that ISG20L1 knockdown decreased levels of autophagic vacuoles and LC3-II after genotoxic stress as assessed by electron microscopy, biochemical, and immunohistochemical measurements of LC3-II.

**Conclusions:**

Our identification of ISG20L1 as a p53 family target and discovery that modulation of this target can regulate autophagic processes further strengthens the connection between p53 signaling and autophagy. Given the keen interest in targeting autophagy as an anticancer therapeutic approach in tumor cells that are defective in apoptosis, investigation of genes and signaling pathways involved in cell death associated with autophagy is critical.

## Background

Recently, several studies have shown that p53 can regulate autophagy in both a transcriptionally-dependent and -independent manner [1]. Autophagy is commonly studied as a mechanism to maintain metabolic homeostasis in cells undergoing starvation [2]. During starvation, cells form double membrane autophagosomes that engulf cellular contents for degradation and these vesicles then recycle the basic metabolic components for consumption [3]. Although originally thought to be primarily induced under conditions of starvation to promote cell survival, autophagy also occurs after various forms of genotoxic stress and plays a role in cell death [4-7]. The role of p53 in DNA damage-induced autophagy is only now being discerned as new reports show a dual role for p53 in the process of autophagy (reviewed in [8,9]). Basal levels of cytoplasmic p53 repress autophagy, a process that increases after the removal or inhibition of p53 [10]. Furthermore, p53 stimulates autophagy through transactivation of target genes such as Sestrins, TSC2, and DRAM (damage-regulated autophagy modulator) (reviewed in [11]). Under conditions of genotoxic stress such as ionizing radiation and camptothecin treatment, p53 has been shown to downregulate mTOR, which lies upstream of ATG-mediated autophagy, through transcriptional regulation of Sestrins1 and Sestrin2 that activate AMPK [12,13]. Upregulated by various stress signals including DNA damage, DRAM is a transcriptional target of p53 that is lysosomal in location and required for p53-induced autophagy, although the direct mechanism by which DRAM regulates autophagy is currently unknown [14].

p63 and p73 are two p53 homologs that share similar structure and have both unique and coordinate roles during development and tumorigenesis [15]. The signaling upstream of each p53 family member is dependent on cellular context and various regulatory mechanisms [reviewed in [16]]. Recently, work from our laboratory has shown that in addition to the interplay of mTOR and p53, inhibition of mTOR activates p73 and results in p73-dependent modulation of genes involved in metabolism and autophagy [16,17]. Though p73 also transcriptionally regulates the p53 target gene DRAM, p73-dependent autophagy does not require DRAM [18].

We have identified numerous, novel candidate p53 target genes by overlaying genes shown to be upregulated after ectopic expression of p53 [19] with genomic loci containing p53 binding sites identified using a ChIP-based yeast one-hybrid screen [20]. Of interest was the discovery of ISG20L1, a gene that was named due to its significant similarity with ISG20L2, a nucleolar protein shown to function in the processing of the 5.8S rRNA [21]. To determine the role that ISG20L1 plays in p53 family signaling, we generated an ISG20L1-specific antibody, analyzed ISG20L1 regulation by all three members of the p53 family, and functionally linked ISG20L1 to genotoxic stress-induced autophagy.

## Results

### ISG20L1 Antibody Production

**Figure 1:**
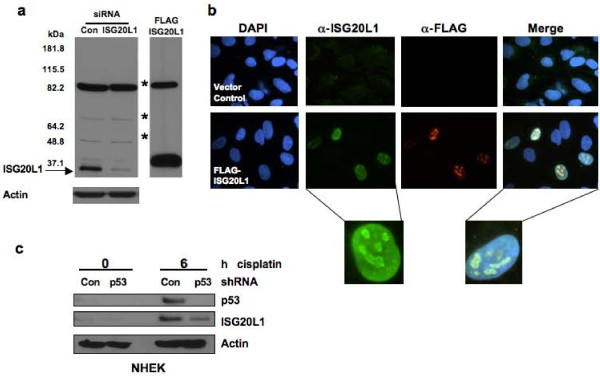
**ISG20L1 antibody production and protein analysis**. **(a) **Western analyses demonstrated the polyclonal antibody generated was able to detect endogenous ISG20L1. H460 cells were reverse transfected with Dharmacon siRNA control (Con) or Dharmacon ISG20L1 Ontarget Plus pool and grown for 72 h, at which point the cultures were treated with 8 Gy ionizing radiation and harvested 24 h after treatment (left panel). Ectopic expression of a FLAG-ISG20L1 detected by our ISG20L1 antibody serves as further positive control. Asterisks mark nonspecific bands and the arrow indicates ISG20L1. Western is representative of three independent experiments. **(b) **ISG20L1 is localized to the nucleolus and perinucleolar region. RKO cells were transfected with either empty vector (Vector Control) or FLAG-ISG20L1 and immunofluorescence analysis was performed 24 h later using a FLAG antibody or the ISG20L1 antibody and the nuclei co-stained with DAPI. Views of individual and merged stainings are shown. Insets are further magnification of individual cells to highlight the perinucleolar staining of ISG20L1. Immunofluorescence was performed on four independent transfections using ISG20L1 and FLAG antibodies. **(c) **p53-dependent regulation of ISG20L1 is shown by Western analysis. NHEK cells were infected with shRNA lentiviral constructs expressing either shRNA to p53 or a scrambled shRNA (Con) and then treated with cisplatin (5 μg/mL) for 6 h. Protein lysates were prepared and analyzed for p53, ISG20L1, and actin. The Western blot is representative of three independent experiments.

The human ISG20L1 gene is 3.1 kb and evolutionarily conserved with 72% identity to *M. musculus*. We generated a rabbit polyclonal antibody to the human ISG20L1 protein (UniProt Q8WTP8) using a 15 amino acid sequence (HGSRGGAREAQDRRN) located at the C-terminus of the protein outside of the exonuclease III domain; database searching confirmed that 100% of these residues are unique to ISG20L1. We performed Western analyses in conjunction with gene overexpression and knockdown assays, to determine that our newly developed antibody could specifically identify a protein of the predicted molecular weight (~37 kD). For overexpression analyses, protein lysates were prepared from H1299 cells engineered to ectopically express FLAG-tagged human ISG20L1. RNA knockdown experiments were performed in H460 cells by reverse transfecting siRNAs directed against ISG20L1 and subsequently treating with ionizing radiation to upregulate endogenous ISG20L1 protein levels (Figure 1a). The antibody we produced had specificity for ISG20L1, the levels of which were significantly reduced after siRNA knockdown or enhanced with ectopic expression of ISG20L1, respectively (Figure 1a). These results are the first demonstration of detection and regulation of endogenous ISG20L1 protein.

Having confirmed antibody specificity, we analyzed the cellular localization of ISG20L1 in H1299 cells ectopically expressing a FLAG-tagged ISG20L1. Immunofluorescence analyses showed nuclear localization of ectopically expressed ISG20L1, similar to the staining pattern seen using a FLAG antibody (Figure 1b). Merging nuclear DAPI staining with ISG20L1-specific staining, showed ISG20L1 localizes to a region of the nucleus having decreased density identified as the nucleolus and higher magnification analyses confirm increased intensity at perinucleolar regions (Figure 1b). Although detectable by Western, we were unable to identify endogenous ISG20L1 using immunofluorescence.

### p53 Family Regulation of ISG20L1

To analyze p53 regulation of ISG20L1 we used primary cultures of normal human keratinocytes (NHEKs), a model system with intact p53 signaling [19,22]. NHEKs were infected with control shRNA or shRNA targeting p53 and exposed for 6 h to cisplatin to elevate p53 activity. Western analysis showed that both p53 and ISG20L1 protein levels were elevated after cisplatin treatment and this increase was primarily p53-dependent as the shRNA targeting p53 significantly decreased the cisplatin-induced elevation in p53 and ISG20L1 protein levels (Figure 1c). We hypothesized that residual ISG20L1 expression was due to cisplatin-mediated elevation of TAp73 activity or protein as previously shown [23-26]. However, p73 protein is difficult to detect in primary cultures of normal human keratinocytes, likely due to the low level of expression in normal cells [19].

**Figure 2:**
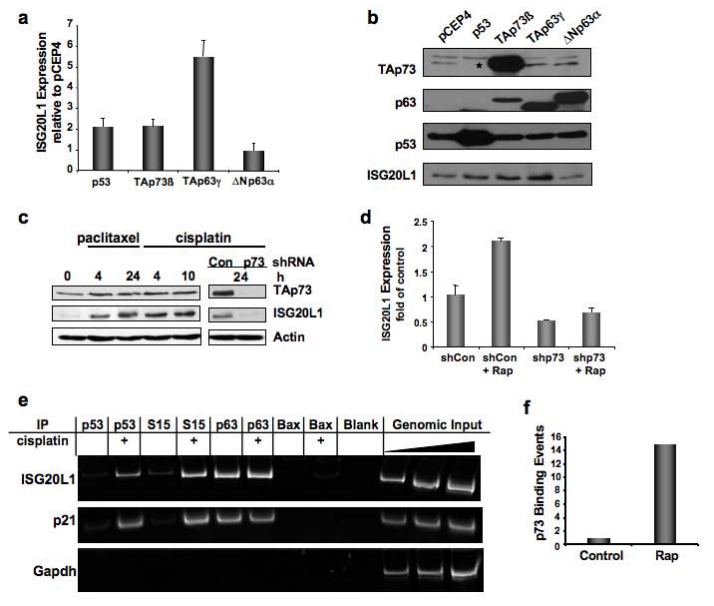
**p53 family regulates expression of ISG20L1**. **(a) **293FT cells were transfected with vector control (pCEP4) or the indicated p53 family members for 24 h and then analyzed by qRT-PCR for ISG20L1. Results represent 3 independent experiments and error bars show standard error. **(b) **Experiment was performed as in (a) and Western analysis performed. Results are representative of 3 independent experiments. *The p63 antibody used cross-reacts with p73 due to sequence similarity in the region containing the epitope recognized by the antibody [65]. **(c) **p73 regulates ISG20L1, seen in Rh30 cells treated with paclitaxel (5 nM) and cisplatin (5 μg/mL) for times indicated. Right panel, Rh30 cells were infected with a pSico vector that expresses control virus (Con) or shRNA targeting all isoforms of p73. Results are representative of 3 independent experiments. **(d) **MDA-MB-231 cells were infected as above. Forty-eight h later, cells were treated with rapamycin (40 nM) and RNA harvested for real-time analyses 24 h after treatment. Results represent 3 independent experiments and error bars show standard error. **(e) **Antibodies specific to p53, p53-Ser15 (S15), p63, and Bax (negative control) were used for ChIP analysis of the ISG20L1 promoter under control and cisplatin-treated (24 h, 10 μg/mL) conditions in HMECs. The p53 binding site in the p21 promoter and a region containing no p53 binding sites in the GAPDH promoter serve as positive and negative controls, respectively. Experiment is representative of duplicate independent templates. **(f) **ChIP analysis of p73 binding to the ISG20L1 promoter in Rh30 cells treated with rapamycin (40 nM) for 24 h.

Given the residual expression of ISG20L1 in p53-depleted keratinocytes (Figure 1c) and the overlapping binding and activity of p53 family members at many regulatory regions in the genome, we hypothesized that ISG20L1 is also regulated by p63 and p73. To test this hypothesis, we transfected 293FT cells with plasmids encoding the transcriptionally active isoforms of the p53 family (p53, TAp73β, and TAp63γ) as well as the transcriptional repressor ΔNp63α. These cells express low levels of TAp73, non-detectable p63, and wild-type p53 that is stabilized and inactivated by association with E1A and large T antigen (see pCEP4 control lane of Figure 2b). Twenty-four h after transfection, we isolated RNA and protein and analyzed ISG20L1 by qRT- PCR and Western, respectively. ISG20L1 levels were increased approximately 2-fold or more by p53, TAp73β, and TAp63γ while ΔNp63α expression decreased levels of ISG20L1 as seen at both the mRNA and protein level (Figure 2a and 2b).

Noting the elevation of ISG20L1 after TAp73 expression, we analyzed the ability of endogenous TAp73 to regulate ISG20L1 using the Rh30 rhabdomyosarcoma cell line. Rh30 cells do not express p63 and contain mutant p53, thereby allowing us to investigate the endogenous regulation of ISG20L1 solely by p73. We treated cells with paclitaxel or cisplatin, two agents known to increase p73 activity [27,28], and observed an elevation in TAp73 protein levels that were accompanied by an increase in ISG20L1 expression (Figure 2c). Elevation of ISG20L1 was TAp73-dependent as shRNA depletion of TAp73 eliminated ISG20L1 expression after treatment (Figure 2c). To verify p73-dependent regulation was not cell-type or damage specific, we infected MDA-MB-231, cells that are also lacking p63 and mutant for p53, with a shRNA lentivirus targeting p73 and treated with rapamycin, an agent known to elevate p73 activity in this cell line [29]. Rapamycin is an inhibitor of the TOR pathway that regulates cell growth and cell cycle progression based on nutrient-dependent signaling and thus rapamycin has similar effects as nutrient starvation [30]. ISG20L1 RNA levels were decreased ~50% by RNAi knockdown of p73, and rapamycin treatment resulted in a greater than 2-fold induction in ISG20L1 expression that was abrogated with p73 knockdown (Figure 2d). Thus, ISG20L1 can be modulated by various forms of cell stress (genotoxic and metabolic), and in the absence of p53 its expression is dependent on other p53 family members.

Next we explored the ability of the p53 family members to bind the ISG20L1 promoter region. Previous findings suggest that the p53 family members have similar transcription factor binding domains, but p53 and p63 have different affinities due to slight differences in consensus site sequence composition and co-factor binding sites present in the promoter regions of regulated genes [31-34]. The p53 binding site discovered by our previous ChIP-based screen, CCACATGCCC-0-GGGCAAGCCC, was located approximately 450 bp upstream of the ISG20L1 transcriptional start site and matches the p53 canonical binding site at 18 of 20 base pairs, with no spacer in the palindrome [20]. To determine if p53 and p63 bind and regulate ISG20L1 at the same promoter region, we used human mammary epithelial cells (HMECs) that express p53 and p63 at levels sufficient for chromatin analyses [32]. HMECs were chemically crosslinked under control and cisplatin-treated conditions, the latter agent can regulate the p53 signaling axis [27,35]. Chromatin was prepared and immunoprecipitated with antibodies to p53, p53-Ser15, p63, and a negative control antibody against a non-DNA binding protein (Bax). Primers were used to amplify the region of the ISG20L1 gene previously reported to contain the p53 binding site [20]. Chromatin immunoprecipitation analysis (ChIP) showed increased binding of p53 and p53-Ser15 after cisplatin treatment, and p63 bound the promoter region of ISG20L1 under both control and cisplatin treated conditions (Figure 2e). These data indicate that both family members cooperate to regulate ISG20L1 expression.

Given that HMECs do not express levels of p73 sufficient for chromatin analysis we performed p73 ChIP in the Rh30 cells to assess p73 binding levels at the ISG20L1 promoter in response to rapamycin treatment. After rapamycin treatment, p73 binding at the p53 consensus binding site in the ISG20L1 promoter increased ~15-fold as compared to a vehicle only-treated control (Figure 2f). Collectively, these data show that all three p53 family members can bind to the promoter region of ISG20L1 and regulate its gene expression.

### ISG20L1 and Cell Death

**Figure 3:**
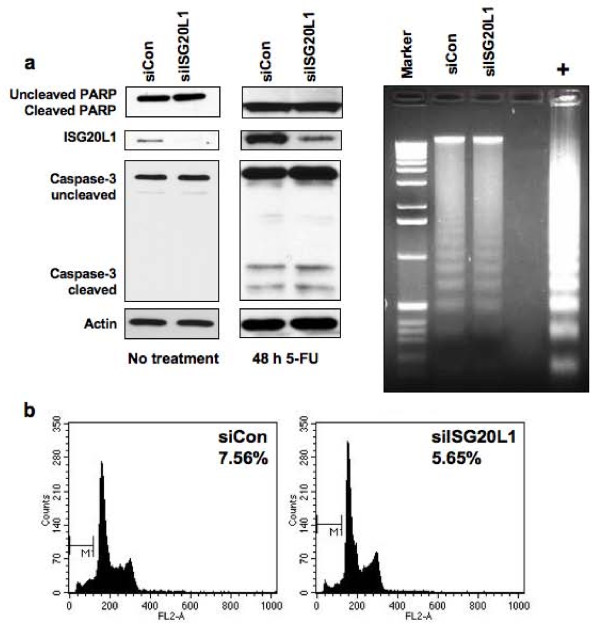
**Knockdown of ISG20L1 does not affect 5-FU-induced apoptotic cell death**. **(a) **RKO colon cancer cells were reverse transfected with control (siCon) or ISG20L1 targeting siRNA. Three days after transfection the cells were treated with 5-FU for 48 h and harvested for Western analysis. The Western blot (left and middle panel) is representative of three independent experiments and shows no change in caspase-3 or PARP cleavage after knockdown of ISG20L1 at either baseline 0 h, where there is low level, basal ISG20L1 expression or after 5-FU treatment. The right panel shows DNA laddering as observed on an ethidium bromide-stained gel, a characteristic marker of apoptosis, from the same samples as above treated with 5-FU. No difference in DNA laddering was evident (lanes are loaded evenly for DNA content). Positive control (+) was obtained from the Roche DNA laddering kit and represent U937 cells treated with camptothecin. **(b) **Flow cytometric analysis was performed on the samples described above at 48 h after 5-FU treatment and sub-G_1 _percentage was calculated.

Shortly after our discovery of ISG20L1 as a p53 target [20], ISG20L1 was reported to have exonuclease function *in vitro *[36] prompting us to determine if it played a role in DNA laddering during the execution phase of apoptosis. Using siRNA knockdown, we decreased ISG20L1 levels in RKO cells and treated with 5-flourouracil (5-FU) to induce apoptosis. Neither knockdown of ISG20L1 nor 5-FU treatment after knockdown affected the onset or extent of apoptosis as measured by analyses of PARP and caspase-3 cleavage, sub-G1 content quantified by flow cytometry, and DNA laddering (Figure 3a and 3b). These data suggest that ISG20L1 does not play a role in the execution phase of apoptosis.

**Figure 4:**
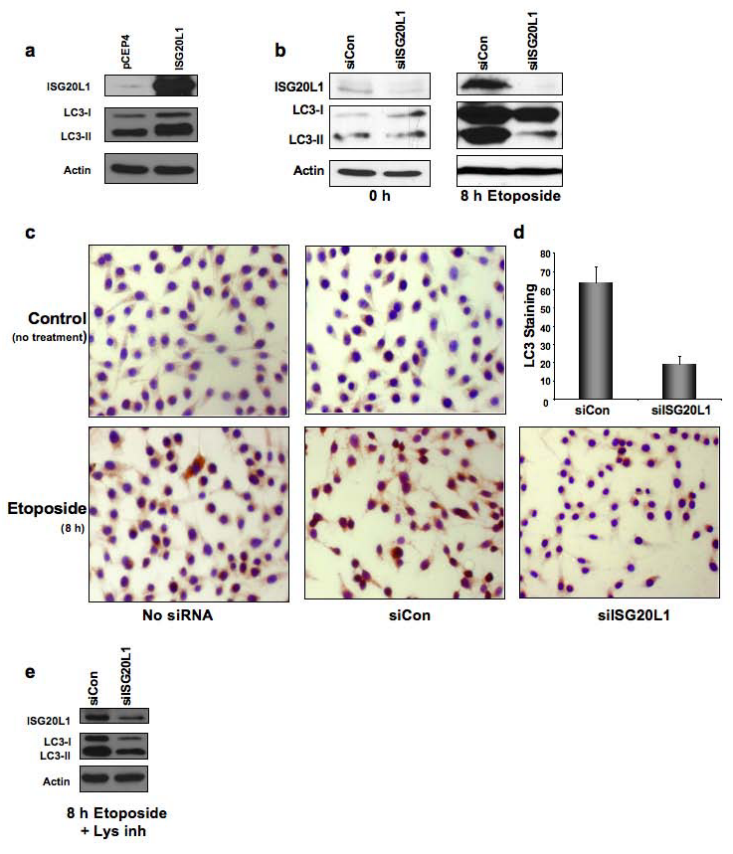
**ISG20L1 expression modulates autophagy**. **(a) **RKO cells were transfected with control, pCEP4, or ISG20L1 for 24 h before harvesting for Western analysis of ISG20L1, LC3, and actin. The Western blot is representative of four independent experiments. **(b) **RKO cells were reverse transfected with control or siISG20L1 and then treated with etoposide (20 μM) for 8 h before harvesting for Western analysis of ISG20L1, LC3, and actin. Western blot is representative of five independent experiments. **(c) **As performed in (b) knockdown of ISG20L1 suppresses autophagy as measured by IHC using an antibody specific to the cleaved form of LC3. IHC analysis of LC3 was performed in RKO cells induced to undergo autophagy after treatment with etoposide (20 μM). Controls include no siRNA and transfection of siRNA alone. **(d) **Results from (c) were quantified by counting number of cells staining positive for LC3 (dark brown) and dividing by the total number of cells (purple nuclear stain) to attain % LC3. Results are representative of 3 independent experiments and error bars represent standard deviation. **(e) **To measure autophagy flux, RKO cells were reverse transfected with control or siISG20L1 and treated with etoposide (20 μM) and lysosomal inhibitors (10 μg/mL E64d and pepstatin A) for 8 h before harvesting for Western analysis of ISG20L1, LC3, and actin. Results are representative of four independent experiments.

To determine if ISG20L1 plays a role in genotoxic stress-induced autophagy we analyzed the effect of ISG20L1 modulation (ectopic expression or knockdown) in RKO cells after etoposide, a treatment that induces autophagy. During autophagy an ubiquitin-like signaling cascade is initiated that results in cleavage of a protein essential for autophagy, microtubule associated-protein 1 light chain 3 (MAP1LC3) (reviewed in [37]). After cleavage and post-translational modification (lipidation), MAP1LC3 (LC3-II) associates with autophagosomal membranes [38], and this modified form of LC3-II is used as a reliable molecular marker of autophagy [39]. We transfected RKO cells with vector control (pCEP4) or pCEP4 expressing ISG20L1. RKO cells ectopically expressing ISG20L1 showed an increase in LC3-II by Western analysis (Figure 4a). Next we reverse transfected RKO cells with control or ISG20L1 siRNA and treated with etoposide. Etoposide treatment resulted in a considerable increase in both ISG20L1 and LC3-II protein levels (Figure 4b, right panel). Robust knockdown of ISG20L1 resulted in a significant reduction in LC3-II as measured by Western (Figure 4b) and an ~70% reduction in LC3 positive cells as measured by immunohistochemistry (IHC) using an antibody that detects endogenous, cleaved LC3 (Figure 4c and 4d). To assess if knockdown of ISG20L1 was modulating autophagy flux, we added protease inhibitors, E64d and pepstatin A, to inhibit lysosomal degradation and LC3-II turnover [39]. RKO cells were treated with etoposide and lysosomal inhibitors for 8 h, three days after reverse transfection with control or ISG20L1 siRNA. Under these conditions, knockdown of ISG20L1 decreased LC3-II levels and thus autophagic flux (Figure 4e).

**Figure 5:**
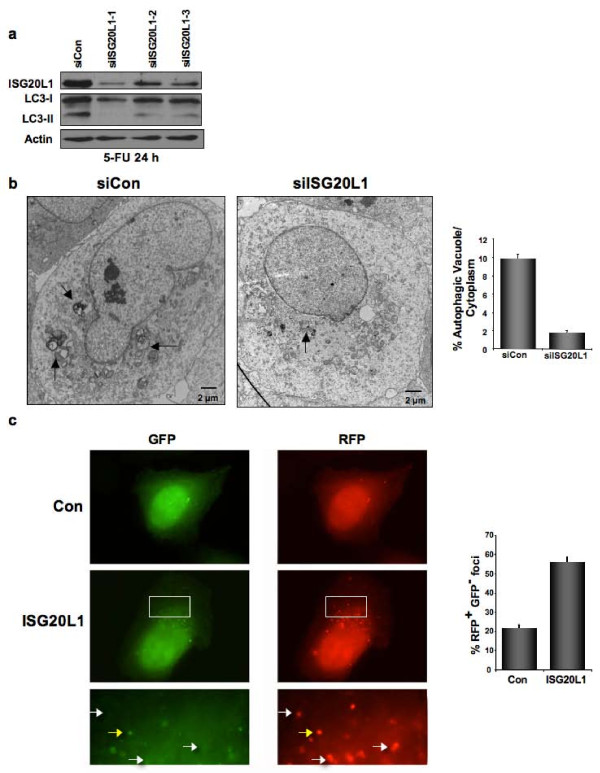
**Genotoxic stress-induced autophagy and autophagy flux is modulated by ISG20L1**. **(a) **U2OS cells were reverse transfected with three unique siRNAs targeting ISG20L1 and treated for 24 h (0.13 mM 5-FU) to induce autophagy as seen in the siControl lane by the modification of LC3-I to LC3-II. Western blot analysis is representative of four independent experiments. **(b) **U2OS cells were reverse transfected with control or siRNA targeting ISG20L1 and 3 days later treated for 24 h with 5-FU. Electron microscopy was performed and representative images can be seen (left panel) Arrowheads indicate autophagosomes or autophagolysosomes. Morphometric analyses was performed on electron micrographs and the percentage of autophagic vacuoles per cytoplasmic volume is shown. Results represent the mean and standard error, p < .0001, n = 25 (right panel). This experiment was performed in duplicate. **(c) **Vector control and ISG20L1 were transfected in U2OS cells stably expressing the tandemly tagged mRFP-GFP-LC3 (tLC3) and then treated for 24 h with 5-FU. Representative panels show GFP alone and RFP alone for control and ISG20L1 as well as magnification of the area outlined by the white box of ISG20L1. White arrows highlight RFP+GFP- only foci representative of autolysosomes (late stage autophagy, RFP^+^GFP^-^); and yellow arrows indicate early autophagosomes where both GFP and RFP are fluorescing (RFP^+^GFP^+^). Quantitation was performed and results are the mean and standard error of RFP^+^GFP^- ^foci expressed as a percentage of total foci (p < .001, n = 50).

To verify these results were not cell type-, damage-, or assay-specific U2OS cells were transfected with control siRNA or three unique siRNAs that target ISG20L1 with varying degrees of knockdown. After treatment with 5-FU, LC3-II levels decreased in a dose-dependent manner relative to levels of ISG20L1 knockdown (Figure 5a). We further determined that knockdown of ISG20L1 in U2OS cells treated with 5-FU does not alter cell cycle distribution (Additional File 1).

Autophagy was first studied and quantified using electron microscopic (EM) detection of autophagosomes [39-41]. To verify that the modulation of LC3-II observed in 5-FU treated U2OS cells was a reliable marker of autophagy, we performed EM on parallel cultures of U2OS cells expressing either control siRNA or the siISG20L1-1 and representative electron micrographs are shown (Figure 5b). Morphometric analysis [42,43] showed an approximately 6-fold decrease in the percentage of autophagic vacuole volume fraction after knockdown of ISG20L1 (Figure 5b, p < 0.0001, n = 25 cells, duplicate experiments).

As described in the previous section, after autophagy induction, lipidated LC3-II is associated with autophagosomal membranes, resulting in the formation of punctate foci that can be quantified by fluorescence microscopy [38,39]. To assess autophagy flux in the U2OS cell system, we used a LC3 (mRFP-GFP-LC3) vector that generates a LC3 fusion protein tagged at the 5' end with red fluorescent protein (RFP) and green fluorescent protein (GFP). Expression of mRFP-GFP-LC3 allows distinction between early autophagic organelles (dual RFP+GFP+ puncta) and mature, acidified autolysosomes (RFP+ GFP-puncta) as the GFP signal is quenched in acidic compartments [39,44]. U2OS cells stably expressing mRFP-GFP-LC3 were transfected with control or ISG20L1 expressing vectors and treated with 5-FU for 24 h. Those cells ectopically expressing ISG20L1 had a greater number of total LC3 foci and a 2.6-fold increase in the percentage of (RFP+GFP-) LC3 puncta per cell representing an increase in maturing autophagosomes (Figure 5c, p < 0.001, n = 50 cells; yellow arrows represent early autophagosomes that are RFP^+^GFP^+^, white arrows indicate late autolysosomal foci that are RFP^+^GFP^-^). These data show that ISG20L1 affects autophagy flux through autophagosome formation and maturation into autolysosomes.

To extend and translate our mechanistic findings to the biologically relevant endpoint of cell growth, we analyzed the effect of ISG20L1 expression using colony formation assays. We transfected RKO, H1299, HCT116 cells as well as ATG5^+/+ ^and ATG5^-/- ^mouse embryonic fibroblasts (MEFs) with control or ISG20L1 expression vectors, selected the cells in hygromycin for 10 days, and measured clonogenic growth. ATG5^-/- ^MEFs were derived from an ATG5-null mouse model system and shown to be autophagy defective [45].

**Figure 6:**
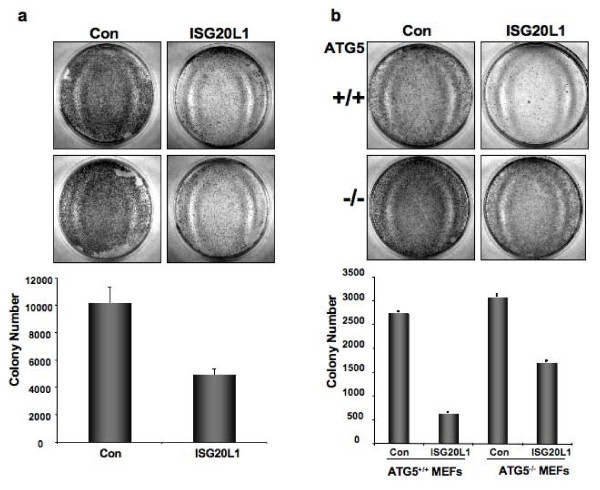
**ISG20L1 decreases cell survival that is partially rescued in ATG5^-/- ^MEFs**. **(a) **HCT116 cells were transfected with pCEP4 vector control or ISG20L1, selected in hygromycin B, and the number of colonies formed after selection were counted using BioRad Quantity One software, shown in the lower panel. Duplicates of each transfection are shown. Similar results were observed in cultures of RKO and H1299 cells (data not shown). Results represent the mean and standard error. **(b) **ATG5^+/+ ^and ATG^-/- ^MEFs were transfected with vector control (pCEP4) and ISG20L1; selected in hygromycin B; and the number of colonies formed after selection were counted using BioRad Quantity One software. Representative pictures are shown (upper panels) for each plasmid in both autophagy proficient (ATG5^+/+^) and deficient (ATG^-/-^) MEFs. Colony number quantified in the lower panel represent mean and standard error of each transfection for both ATG5^+/+ ^and ATG^-/- ^MEFs performed in triplicate.

A representative result from one of the tumor-derived cell lines (HCT116) is presented in Figure 6a. Cells ectopically expressing ISG20L1 had a 48% reduction in colony formation as compared to those cultures expressing an empty vector control. Parallel flow cytometric analyses were performed at 48, 72, and 96 h after transfection and no differences were observed in sub-G_1 _DNA content or Annexin V staining, between control and ISG20L1 expressing cells (Additional File 2a and 2b). Use of the ATG5^+/+ ^and ATG5^-/- ^MEFs enabled us to determine if the decreased clonogenic survival after expression of ISG20L1 was dependent on ATG5-induced autophagic processes. As observed in the human cell lines, ectopic expression of ISG20L1 in the ATG5^+/+ ^MEFs decreased colony number by ~77% compared to control. Importantly, this ISG20L1-induced decrease in colony number was partially rescued in ATG5^-/- ^cells (over 2-fold increase; Figure 6b). Collectively, these data are consistent with a function for ISG20L1 in genotoxic stress-induced autophagy and decreased cell survival.

## Discussion

Several studies provide evidence for a role of p53 in autophagy, a process first recognized as important in cell survival and now thought to function in tumor suppression [14,46,47]. We strengthen this link between the p53 signaling axis and genotoxic-stress induced autophagy by identifying ISG20L1 as a transcriptional target of all three p53 family members. Using a newly generated antibody, we show that ISG20L1 levels increase in a p53- and TAp73-dependent manner after various forms of stress. In addition to p53, the family members p63 and p73 can bind and directly regulate ISG20L1 expression. Ectopic expression of ISG20L1 decreased cell survival without induction of apoptosis as determined by flow cytometric analyses of sub-G_1 _DNA content or Annexin V staining, and the decreased clonogenic survival was partly rescued in an autophagy deficient background (ATG5^-/- ^MEFs). ISG20L1 was not involved in modulating 5-FU-mediated apoptosis, as suppression of ISG20L1 in RKO cells did not alter the incidence or extent of apoptosis as measured by PARP and caspase-3 cleavage, sub-G1 content, and DNA laddering. In contrast, siRNA knockdown of ISG20L1 decreased genotoxic stress-induced autophagy as measured by electron microscopy, biochemical, and immunohistochemical analyses of LC3-II. Thus, we identified ISG20L1 as a p53-family dependent, genotoxic stress-induced modulator of autophagy.

The nucleolus is the cellular site of rRNA synthesis and processing as well as ribosomal assembly [48]. One of the first connections of p53 to nucleolar signaling was the observation that a dominant-negative form of the nucleolar protein Bop1 could induce p53-dependent cell cycle arrest [49]. Recent publications have linked nucleolar proteins to arbitrating cellular response to stress, including autophagy [50-52]. For example, nucleolar ARF can inhibit the production of the immature 12S rRNA intermediate, interact with the 5.8S rRNA [53], and activate autophagy in p53-positive cells [54].

Our data validates previous findings of ISG20L1 nucleolar localization [36,55]. ISG20L2, a family member of ISG20L1, also localizes to the nucleolus and is involved in the processing of 12S rRNA to the mature 5.8S rRNA, part of the large ribosomal subunit [21]. *In vitro *assays have shown that the exonuclease III domain of ISG20L1 is required to degrade single- and double-stranded DNA and RNA [36,55]. Collectively, the recent findings that ISG20L1 can degrade RNA, our data and others showing nucleolar localization of ISG20L1, and our linkage of ISG20L1 to autophagy suggests it will be important to examine the role of ISG20L1 in rRNA processing and ribosomal assembly during cellular response to stress [36,55,56].

There is growing evidence for the interplay between autophagy and the p53 family. As mentioned above, p19ARF and the short mitochondrial form (smARF) are able to induce autophagy in both p53-dependent and -independent manners [54]. A number of genes involved in autophagy are directly regulated by p53 including the mTOR inhibitors, TSC1 and PTEN, Sestrin1 and Sestrin2, and the damage-regulated autophagy modulator (DRAM) [14,46]. Additionally, inhibition of mTOR by p53 is associated with autophagy and occurs through DNA damaged-induced signaling involving AMPK and TSC1/2 [46]. p73 transcriptional activity has also been linked to autophagy as p73 is bound to a number of genes involved in metabolism and autophagy [17,18]. Our results show that ISG20L1 is contributing to cellular demise by modulating the process of autophagy that is commonly associated with type II cell death [57,58].

## Conclusion

The identification of ISG20L1 as a p53 family target and discovery that modulation of this target can regulate autophagic processes further strengthens the connection between p53 signaling and autophagy. Given the keen interest in targeting autophagy as an anticancer therapeutic approach in tumor cells that are defective in apoptosis, investigation of genes and signaling pathways involved in cell death associated with autophagy is critical.

## Methods

### Cell Culture and Treatment

The RKO, U2OS, H460, 293FT, HCT116, and H1299 cell lines were obtained from ATCC and cultured in DMEM medium with 10% fetal bovine serum supplement and 1% penicillin-streptomycin. The ATG5^+/+ ^and ATG5^-/- ^MEFs were a kind gift from Dr. Mizushima (Tokyo Medical and Dental University) and cultured in DMEM medium with 10% fetal bovine serum [46]. The MDA-MB-231 was also obtained from ATCC and cultured in McCoy's 5A medium. The Rh30 cell line was kindly given by Peter Houghton (St. Jude Children's Research Hospital) and cultured in RPMI medium with 10% fetal bovine serum. Normal human epidermal keratinocytes (NHEKs) were obtained from the Vanderbilt Skin Disease Research Core and cultured as previously described [59]. Primary human mammary epithelial cells (HMECs) were purified from normal breast tissue obtained by the Vanderbilt-Ingram Cancer Center Human Tissue Acquisition and Pathology Shared Resource Core, and were isolated and grown as previously described [22,60].

The following chemotherapeutics were used in treatment of cell lines mentioned above as described in results 8 Gy ^137^Cs ionizing radiation, 0.13 mM 5-FU (APP Pharmaceuticals), 20 μM etoposide (Bedford Laboratories), 5 μg/mL cisplatin (APP Pharmaceuticals), 5 nM paclitaxel (Sigma), 40 nM rapamycin (Calbiochem). Lysosomal inhibitors were used at final concentration of 10 μg/mL of E64d (Calbiochem 330005) and pepstatin A (MP Biomedical 195368).

### Cell Transfection and Small Interfering RNA

The following targeting sense strand sequences were used for siRNA: Dharmacon siControl (Non-Targeting siRNA #1) UAGCGACUAAACACUCAA; Dharmacon siISG20L1-1 CAGCAAGGUUCACGGAUAUUU; siISG20L1-2, AUACUAAGCAAGCGAGGGAUU; siISG20L1-3, CUCAAUUGGAAACGUGAAAUU. Dharmacon siRNA ISG20L1 pools consisted of the above targeting vectors plus siISG20L1-4 CAGCAGGGCCACUCGUCUA. Dharmacon siRNAs were reverse transfected into H460, U2OS, and RKO cells (4.5 × 10^5^) with Lipofectamine2000 (Invitrogen) according to the manufacturer's protocol.

To knockdown p53 in NHEK cells, a 19-bp short hairpin RNA, corresponding to nucleotides 611 to 629 of p53 RNA (GenBank NM000546), was annealed and cloned into the self-inactivating lentiviral vector (H1-LV) that contains a GFP reporter gene under control of human ubiquitin C promoter for monitoring infection efficiency. A scrambled oligonucleotide was designed as a negative control and also cloned in the H1-LV vector. These lentiviral vectors were transfected using CaPO_4 _methods into 293FT cells. After 48 h viral medium was harvested and with the addition of 8 μg/mL polybrene used to infect NHEK cells.

293FT cells were transfected using Fugene 6 (Roche) to make pSico lentivirus. To knockdown p73 in MDA-MB-231 and Rh30, cells were infected with the pSico lentivirus system that expresses shRNA targeting all isoforms of p73 as previously described [29]. Forty-eight h later, cells were treated with rapamycin (40 nM) and RNA harvested 24 h later.

293FT cells were transfected using Lipofectamine2000 with either pCEP4 empty control or cDNAs encoding p53, TAp63γ, TAp73β, or ΔNp63α and harvested 24 h later for RT-PCR or Western analysis.

Clonogenic Survival Assays were performed in HCT116, RKO, H1299 cells, as well as ATG5^+/+ ^and ATG5^-/- ^MEFs transformed with SV40 large T antigen obtained from Dr. Mizushima [45]. For all cell lines, Lipofectamine2000 was used to transfect either pCEP4 empty vector control or ISG20L1 in 60 mm dishes. Twenty-four h after transfection, cells were selected for 10 days under the appropriate hygromycin B concentration determined per cell line. Colonies were Wright stained and analyzed using the Biorad Quantity One software.

### Western Analysis and Antibodies

Western analyses were performed as previously described [61]. Fourteen percent SDS-polyacrylamide gels were used for analysis of LC3 using anti-MAP1LC3-II (Abgent AP1802a). Additional antibodies used for protein detection: anti-p53 (Santa Cruz Biotechnology, PAb1801), anti-β-Actin (Sigma-Aldrich, A5441-0.2 mL), anti-PARP (Cell Signaling, #9542), anti-Caspase-3 (Cell Signaling, #9662), anti-p73 (Bethyl A300), p63 (4A4) (Santa Cruz, sc-8431), and anti-ISG20L1 (Bethyl Laboratories, rabbit affinity purified antibody). A peptide for ISG20L1 antibody production was designed at the C-terminus of ISG20L1, outside of the functional exonuclease domain found from amino acids 111-275, with the intent to increase antigenicity and accessibility of the antibody while decreasing possible cross-reactivity. The peptide product sequence "HGSRGGAREAQDRRN" targets amino acids 311-325 of ISG20L1 and these 15 amino acids are unique to the ISG20L1 sequence.

### RNA Isolation and Real-Time Analysis

RNA isolation and all subsequent quantitative real-time PCR (qRT-PCR) analyses were performed as described previously [20]. All primer sets were run under the following cycling conditions: 95°C for 3 minutes followed by 40 cycles of: 95°C for 10 sec and annealing at 60°C for 45 sec, with data acquisition during each cycle. Melting curve analysis following PCR cycling was used to determine purity and quality of PCR product.

### Immunofluorescence, Immunohistochemistry, and Electron Microscopy

For immunofluorescence analysis, cells were grown on glass coverslips and fixed in a 4% paraformaldehyde solution for 10 min at room temperature. After rinsing with PBS, the cells were permeabilized with 0.5% Triton X-100 for 10 min. Following another rinse with PBS, cells were blocked for 15 min at room temperature with 5% BSA-PBS solution. The ISG20L1 (Bethyl) and FLAG antibodies (Sigma, F3165 anti-FLAG M2) were diluted in 1% BSA-PBS and incubated on cells at 37°C with 5% CO_2 _for 1 h. The coverslips were washed 3× with PBS and placed in 2° rabbit anti-Alexa Flour 546 and mouse anti-Alexa Flour 488, respectively for 1 h at room temperature, in the dark. The cells were washed 3× with PBS and counterstained with DAPI. All images were obtained using 1000× magnification on a Zeiss Axioplan microscope equipped with a Zeiss camera and software.

Direct immunofluorescence was performed on U2OS cells stably expressing mRFP-GFP-LC3. The mRFP-GFP-LC3 expression vector was kindly provided by Dr. Yoshimori (Osaka University) [44] and Dr. Mizushima (Tokyo Medical and Dental University) [38]. U2OS stably expressing the tagged LC3 protein were generated by transfecting the cells with the mRFP-GFP-LC3 expression vector using FuGENE 6 (Roche, Indianapolis, IN) and selecting in geneticin (Cellgro, Manassas, VA). Engineered U2OS cells were then transfected with either pCEP4 control or ISG20L1 expression plasmids and treated for 24 h with 5-FU. The cells were fixed and analyzed as above using a Zeiss Axioplan. Fifty cells were counted, without knowledge of the plasmids expressed, and RFP-only foci are reported as a percentage of total foci.

For immunohistochemistry analysis, cells were grown on glass coverslips. The cells were fixed, and permeabilized as indicated above for IF analysis. Washes were done in 1× TBS/0.1% Tween-20 (1× TBST), and cells were blocked overnight rocking at 4°C in 5% normal goat serum diluted in TBST. The coverslips were stained specifically for the cleaved LC3 using the Abgent LC3 specific 1° antibody (Abgent AP1806a) for 30 min at room temperature. The coverslips were then washed 3 times in TBST. The secondary used was the Dako Cytomation LSAb2 system HRP kit (K0673) according to manufacturer's protocol. Cells were analyzed for LC3 staining and counted at 200× magnification.

U2OS cells were reverse transfected using Lipofectamine2000 with Dharmacon Nonsilencing control or siRNA targeting ISG20L1. Three days after reverse transfection, cells were treated or not for 24 h with 5-FU to induce autophagy. Cells were harvested, washed with PBS, and exposed to 2% glutaraldehyde for fixation. Sample were rinsed in buffer, postfixed in 1% OsO_4 _for 1 h, dehydrated through an ethanol series and transferred into Epon resin. Ultrathin sections (60-70 nm, silver-gray) were obtained using a Reichert Ultracut E microtome with a diamond knife, transferred to formvar-coated grids, and examined on a Phillips CM-10 transmission electron microscope (FEI, Hillsboro, OR), operating at 80 kV, and images were captured with an AMT 2 mega pixel camera (Advanced Microscopy Techniques, Danvers, MA).

Two replicates were performed and each time 25 micrographs were counted blindly for each control and ISG20L1 knockdown. Additionally, cells were photographed in an un-biased fashion according to their placement on the grid. Images were quantified using ImageJ software and taking into account various acceptable methods [39,42]. We set to scale the pixel ratio to microns and used measurement analysis to quantify the area occupied by autophagosome and autolysosomes as compared to the total cytoplasmic area excluding the nucleus. Autophagosomes were defined as double or multiple membrane structures surrounding cytoplasmic material, and autolysosomes were defined as single membrane structures surrounding cytoplasmic constituents at various levels of degradation [62].

### Flow Cytometric Analyses

Flow cytometry was performed as previously described using a FACSCaliber instrument (Becton-Dickinson) [63]. Annexin V-FITC staining detected by flow cytometry was performed using the Annexin V-FITC apoptosis detection kit (BD Pharmingen, 556547).

### Chromatin Immunoprecipitation Analyses

HMECs were treated or not with 10 ug/mL cisplatin for 24 h and chromatin was prepared as previously described [64]. PCR amplification was performed using primers ISG20L1 forward CAGCCTGTCCAACATGGC and ISG20L1 reverse GCTGAGGCCATAACTTGGAAA, GAPDH forward CACCAGCCATCCTGTCCTCC and GAPDH reverse GTTCCTTCCCAGCCCCCACT, and p21 forward GCTTGGGCAGCAGGCTG and p21 reverse AGCCCTGTCGCAAGGATC as previously described [19]. PCR was performed using one cycle of 5 min at 95°C; followed by different number of cycles as indicated below of: 95° for 30 s, annealing temperature as indicated below for 45 s, and 30 sec of 72°C; to be finished with 10 min at 72°C. AEN 40 Cycles Anneal 54°C, GAPDH 35 Cycles Anneal 62°C, and p21 35 Cycles Anneal 57°C. Amplified DNA was resolved on a 6% polyacrylamide gel and stained after with ethidium bromide.

To attain sufficient levels of p73 for ChIP analysis, ~1.7 × 10^7^rapidly growing Rh30 cells were treated for 24 h using vehicle control or 40 nM rapamycin. The samples were prepared and Genpathway analysis performed as previously described [17] using the p73 antibody (Bethyl Laboratories, A300) for immunoprecipitation.

### DNA Laddering

Cells were counted and 2 × 10^6 ^cells were removed and washed in PBS for DNA laddering analysis. Procedure was followed according to the Roche Apoptotic DNA-Ladder Kit (11 835 246 001). In brief, cells were lysed in an equal volume of proprietary lysis buffer, incubated for 10 min at room temperature, 100 μl of isopropanol was added and vortexed prior to loading the sample onto filter tubes. Filter tubes were spun 2× 1 min at 8000 rpm and washed after each spin with 500 μl washing buffer. After discarding flow through, filter tube samples were placed in collection tubes and 100 μl elution buffer was added and then spun for 1 min at 8000 rpm. DNA obtained from samples was run on a 1% agarose gel next to 1 kb DNA ladder and positive control DNA (U937 cells treated with camptothecin) supplied from Roche.

### Statistical Analysis

Data were analyzed where indicated using the Student's *t *test for statistical significance. *P *values are indicated in the figure legends and text. Standard deviation and error were calculated and represented in bar graphs where indicated.

## Abbreviations

TSC2: tuberous sclerosis protein 2; mTOR: mammalian target of rapamycin; ISG20L1: interferon stimulated gene 20- like 1.

## Competing interests

The authors declare that they have no competing interests.

## Authors' contributions

KGE designed and performed experiments, analyzed and interpreted data, and prepared the manuscript. JMR, DJM, CBM, CEB, SS, LT, and KNJ performed experiments. JMR also contributed to experimental design. JAP designed experiments, analyzed and interpreted data, and prepared the manuscript. All authors read and approved the final manuscript.

## Supplementary Material

Additional file 1 **Knockdown of ISG20L1 does not alter cell cycle**. **(a) **U2OS cells were reverse transfected with nonsilencing control or siRNA targeting ISG20L1 and three days later treated or not with 5-FU over the indicated timecourse. Western analysis was performed to measure ISG20L1 and actin. **(b) **Flow cytometry was performed for each condition over the timecourse performed in (a) and a representative example of three independent experiments is shown. Click here for file

Additional file 2 **The decrease in clonogenic survival after ectopic ISG20L1 expression is not accompanied by increased apoptosis**. **(a) **Flow cytometry was performed over a timecourse with H1299 cells ectopically expressing either vector control or ISG20L1. A representative example of three separate experiments is shown. **(b) **The sub-G_1_percentage was analyzed for those samples described in part (a). **(c) **To further assess apoptosis, Annexin-V staining and flow cytometry were performed on H1299 cells 48 h after transfection with vector control or ISG20L1 and percent of cells stained for Annexin-V under both experimental conditions shown from three experiments. The error bars represent standard deviation. Click here for file

## References

[B1] Crighton D Wilkinson S Ryan KM DRAM links autophagy to p53 and programmed cell death Autophagy 2007 3 72 74 17102582 10.4161/auto.3438

[B2] Komatsu M Waguri S Ueno T Iwata J Murata S Tanida I Ezaki J Mizushima N Ohsumi Y Uchiyama Y Impairment of starvation-induced and constitutive autophagy in Atg7-deficient mice J Cell Biol 2005 169 425 434 10.1083/jcb.200412022 15866887 PMC2171928

[B3] Levine B Eating oneself and uninvited guests: autophagy-related pathways in cellular defense Cell 2005 120 159 162 15680321 10.1016/j.cell.2005.01.005

[B4] Green DR Chipuk JE p53 and metabolism: Inside the TIGAR Cell 2006 126 30 32 10.1016/j.cell.2006.06.032 16839873

[B5] Kang C You YJ Avery L Dual roles of autophagy in the survival of Caenorhabditis elegans during starvation Genes Dev 2007 21 2161 2171 10.1101/gad.1573107 17785524 PMC1950855

[B6] Scott RC Juhasz G Neufeld TP Direct induction of autophagy by Atg1 inhibits cell growth and induces apoptotic cell death Curr Biol 2007 17 1 11 10.1016/j.cub.2006.10.053 17208179 PMC1865528

[B7] Shimizu S Kanaseki T Mizushima N Mizuta T Arakawa-Kobayashi S Thompson CB Tsujimoto Y Role of Bcl-2 family proteins in a non-apoptotic programmed cell death dependent on autophagy genes Nat Cell Biol 2004 6 1221 1228 10.1038/ncb1192 15558033

[B8] Levine B Abrams J p53: The Janus of autophagy? Nat Cell Biol 2008 10 637 639 10.1038/ncb0608-637 18521069 PMC2739720

[B9] Green DR Kroemer G Cytoplasmic functions of the tumour suppressor p53 Nature 2009 458 1127 1130 10.1038/nature07986 19407794 PMC2814168

[B10] Tasdemir E Maiuri MC Galluzzi L Vitale I Djavaheri-Mergny M D'Amelio M Criollo A Morselli E Zhu C Harper F Regulation of autophagy by cytoplasmic p53 Nat Cell Biol 2008 10 676 687 10.1038/ncb1730 18454141 PMC2676564

[B11] Vousden KH Ryan KM p53 and metabolism Nat Rev Cancer 2009 9 691 700 10.1038/nrc2715 19759539

[B12] Braunstein S Badura ML Xi Q Formenti SC Schneider RJ Regulation of protein synthesis by ionizing radiation Mol Cell Biol 2009 29 5645 5656 10.1128/MCB.00711-09 19704005 PMC2772731

[B13] Budanov AV Karin M p53 target genes sestrin1 and sestrin2 connect genotoxic stress and mTOR signaling Cell 2008 134 451 460 10.1016/j.cell.2008.06.028 18692468 PMC2758522

[B14] Crighton D Wilkinson S O'Prey J Syed N Smith P Harrison PR Gasco M Garrone O Crook T Ryan KM DRAM, a p53-induced modulator of autophagy, is critical for apoptosis Cell 2006 126 121 134 10.1016/j.cell.2006.05.034 16839881

[B15] Murray-Zmijewski F Lane DP Bourdon JC p53/p63/p73 isoforms: an orchestra of isoforms to harmonise cell differentiation and response to stress Cell Death Differ 2006 13 962 972 10.1038/sj.cdd.4401914 16601753

[B16] Rosenbluth JM Pietenpol JA The jury is in: p73 is a tumor suppressor after all Genes Dev 2008 22 2591 2595 10.1101/gad.1727408 18832062

[B17] Rosenbluth JM Pietenpol JA mTOR regulates autophagy-associated genes downstream of p73 Autophagy 2009 5 114 116 10.4161/auto.5.1.7294 19001857 PMC2792737

[B18] Crighton D O'Prey J Bell HS Ryan KM p73 regulates DRAM-independent autophagy that does not contribute to programmed cell death Cell Death Differ 2007 14 1071 1079 10.1038/sj.cdd.4402108 17304243

[B19] Schavolt KL Pietenpol JA p53 and Delta Np63 alpha differentially bind and regulate target genes involved in cell cycle arrest, DNA repair and apoptosis Oncogene 2007 26 6125 6132 10.1038/sj.onc.1210441 17404570

[B20] Hearnes JM Mays DJ Schavolt KL Tang L Jiang X Pietenpol JA Chromatin immunoprecipitation-based screen to identify functional genomic binding sites for sequence-specific transactivators Mol Cell Biol 2005 25 10148 10158 10.1128/MCB.25.22.10148-10158.2005 16260627 PMC1280257

[B21] Coute Y Kindbeiter K Belin S Dieckmann R Duret L Bezin L Sanchez JC Diaz JJ ISG20L2, a novel vertebrate nucleolar exoribonuclease involved in ribosome biogenesis Mol Cell Proteomics 2008 7 546 559 18065403 10.1074/mcp.M700510-MCP200

[B22] Flatt PM Price JO Shaw A Pietenpol JA Differential cell cycle checkpoint response in normal human keratinocytes and fibroblasts Cell Growth Differ 1998 9 535 543 9690621

[B23] Agami R Blandino G Oren M Shaul Y Interaction of c-Abl and p73alpha and their collaboration to induce apoptosis Nature 1999 399 809 813 10.1038/21697 10391250

[B24] Gong JG Costanzo A Yang HQ Melino G Kaelin WG Jr Levrero M Wang JY The tyrosine kinase c-Abl regulates p73 in apoptotic response to cisplatin-induced DNA damage Nature 1999 399 806 809 10.1038/21690 10391249

[B25] Lapi E Iovino A Fontemaggi G Soliera AR Iacovelli S Sacchi A Rechavi G Givol D Blandino G Strano S S100A2 gene is a direct transcriptional target of p53 homologues during keratinocyte differentiation Oncogene 2006 25 3628 3637 10.1038/sj.onc.1209401 16449968

[B26] Yuan ZM Shioya H Ishiko T Sun X Gu J Huang YY Lu H Kharbanda S Weichselbaum R Kufe D p73 is regulated by tyrosine kinase c-Abl in the apoptotic response to DNA damage Nature 1999 399 814 817 10.1038/21704 10391251

[B27] Leong CO Vidnovic N DeYoung MP Sgroi D Ellisen LW The p63/p73 network mediates chemosensitivity to cisplatin in a biologically defined subset of primary breast cancers J Clin Invest 2007 117 1370 1380 10.1172/JCI30866 17446929 PMC1849987

[B28] Oh YK Lee HJ Jeong MH Rhee M Mo JW Song EH Lim JY Choi KH Jo I Park SI Role of activating transcription factor 3 on TAp73 stability and apoptosis in paclitaxel-treated cervical cancer cells Mol Cancer Res 2008 6 1232 1249 10.1158/1541-7786.MCR-07-0297 18644986 PMC3783268

[B29] Rosenbluth JM Mays DJ Pino MF Tang LJ Pietenpol JA A gene signature-based approach identifies mTOR as a regulator of p73 Mol Cell Biol 2008 28 5951 5964 10.1128/MCB.00305-08 18678646 PMC2547001

[B30] Peng T Golub TR Sabatini DM The immunosuppressant rapamycin mimics a starvation-like signal distinct from amino acid and glucose deprivation Mol Cell Biol 2002 22 5575 5584 10.1128/MCB.22.15.5575-5584.2002 12101249 PMC133939

[B31] Ortt K Sinha S Derivation of the consensus DNA-binding sequence for p63 reveals unique requirements that are distinct from p53 FEBS Lett 2006 580 4544 4550 10.1016/j.febslet.2006.07.004 16870177

[B32] Perez CA Ott J Mays DJ Pietenpol JA p63 consensus DNA-binding site: identification, analysis and application into a p63MH algorithm Oncogene 2007 26 7363 7370 10.1038/sj.onc.1210561 17563751

[B33] Shikama N Lee CW France S Delavaine L Lyon J Krstic-Demonacos M La Thangue NB A novel cofactor for p300 that regulates the p53 response Mol Cell 1999 4 365 376 10.1016/S1097-2765(00)80338-X 10518217

[B34] Zhu J Jiang J Zhou W Chen X The potential tumor suppressor p73 differentially regulates cellular p53 target genes Cancer Res 1998 58 5061 5065 9823311

[B35] Fritsche M Haessler C Brandner G Induction of nuclear accumulation of the tumor-suppressor protein p53 by DNA-damaging agents Oncogene 1993 8 307 318 8426740

[B36] Lee JH Koh YA Cho CK Lee SJ Lee YS Bae S Identification of a novel ionizing radiation-induced nuclease, AEN, and its functional characterization in apoptosis Biochem Biophys Res Commun 2005 337 39 47 10.1016/j.bbrc.2005.08.264 16171785

[B37] Cecconi F Levine B The role of autophagy in mammalian development: cell makeover rather than cell death Dev Cell 2008 15 344 357 10.1016/j.devcel.2008.08.012 18804433 PMC2688784

[B38] Kabeya Y Mizushima N Ueno T Yamamoto A Kirisako T Noda T Kominami E Ohsumi Y Yoshimori T LC3, a mammalian homologue of yeast Apg8p, is localized in autophagosome membranes after processing Embo J 2000 19 5720 5728 10.1093/emboj/19.21.5720 11060023 PMC305793

[B39] Klionsky DJ Abeliovich H Agostinis P Agrawal DK Aliev G Askew DS Baba M Baehrecke EH Bahr BA Ballabio A Guidelines for the use and interpretation of assays for monitoring autophagy in higher eukaryotes Autophagy 2008 4 151 175 18188003 10.4161/auto.5338 PMC2654259

[B40] Mizushima N Methods for monitoring autophagy Int J Biochem Cell Biol 2004 36 2491 2502 10.1016/j.biocel.2004.02.005 15325587

[B41] Mizushima N Yoshimori T Levine B Methods in mammalian autophagy research Cell 140 313 326 10.1016/j.cell.2010.01.028 20144757 PMC2852113

[B42] Swanlund JM Kregel KC Oberley TD Investigating autophagy: Quantitative morphometric analysis using electron microscopy Autophagy 6 19923921 10.4161/auto.6.2.10439 PMC3235050

[B43] Yla-Anttila P Vihinen H Jokitalo E Eskelinen EL Monitoring autophagy by electron microscopy in Mammalian cells Methods Enzymol 2009 452 143 164 full_text 19200881 10.1016/S0076-6879(08)03610-0

[B44] Kimura S Noda T Yoshimori T Dissection of the autophagosome maturation process by a novel reporter protein, tandem fluorescent-tagged LC3 Autophagy 2007 3 452 460 17534139 10.4161/auto.4451

[B45] Kuma A Hatano M Matsui M Yamamoto A Nakaya H Yoshimori T Ohsumi Y Tokuhisa T Mizushima N The role of autophagy during the early neonatal starvation period Nature 2004 432 1032 1036 10.1038/nature03029 15525940

[B46] Feng Z Zhang H Levine AJ Jin S The coordinate regulation of the p53 and mTOR pathways in cells Proc Natl Acad Sci USA 2005 102 8204 8209 10.1073/pnas.0502857102 15928081 PMC1142118

[B47] Yue Z Jin S Yang C Levine AJ Heintz N Beclin 1, an autophagy gene essential for early embryonic development, is a haploinsufficient tumor suppressor Proc Natl Acad Sci USA 2003 100 15077 15082 10.1073/pnas.2436255100 14657337 PMC299911

[B48] Scheer U Hock R Structure and function of the nucleolus Curr Opin Cell Biol 1999 11 385 390 10.1016/S0955-0674(99)80054-4 10395554

[B49] Pestov DG Strezoska Z Lau LF Evidence of p53-dependent cross-talk between ribosome biogenesis and the cell cycle: effects of nucleolar protein Bop1 on G(1)/S transition Mol Cell Biol 2001 21 4246 4255 10.1128/MCB.21.13.4246-4255.2001 11390653 PMC87085

[B50] David-Pfeuty T Potent inhibitors of cyclin-dependent kinase 2 induce nuclear accumulation of wild-type p53 and nucleolar fragmentation in human untransformed and tumor-derived cells Oncogene 1999 18 7409 7422 10.1038/sj.onc.1203103 10602500

[B51] Olson MO Sensing cellular stress: another new function for the nucleolus? Sci STKE 2004 2004 pe10 10.1126/stke.2242004pe10 15026578

[B52] Rubbi CP Milner J Disruption of the nucleolus mediates stabilization of p53 in response to DNA damage and other stresses Embo J 2003 22 6068 6077 10.1093/emboj/cdg579 14609953 PMC275437

[B53] Sugimoto M Kuo ML Roussel MF Sherr CJ Nucleolar Arf tumor suppressor inhibits ribosomal RNA processing Mol Cell 2003 11 415 424 10.1016/S1097-2765(03)00057-1 12620229

[B54] Abida WM Gu W p53-Dependent and p53-independent activation of autophagy by ARF Cancer Res 2008 68 352 357 10.1158/0008-5472.CAN-07-2069 18199527 PMC3737745

[B55] Kawase T Ichikawa H Ohta T Nozaki N Tashiro F Ohki R Taya Y p53 target gene AEN is a nuclear exonuclease required for p53-dependent apoptosis Oncogene 2008 27 3797 3810 10.1038/onc.2008.32 18264133

[B56] Kraft C Deplazes A Sohrmann M Peter M Mature ribosomes are selectively degraded upon starvation by an autophagy pathway requiring the Ubp3p/Bre5p ubiquitin protease Nat Cell Biol 2008 10 602 610 10.1038/ncb1723 18391941

[B57] Eisenberg-Lerner A Bialik S Simon HU Kimchi A Life and death partners: apoptosis, autophagy and the cross-talk between them Cell Death Differ 2009 16 966 975 10.1038/cdd.2009.33 19325568

[B58] Bursch W Karwan A Mayer M Dornetshuber J Frohwein U Schulte-Hermann R Fazi B Di Sano F Piredda L Piacentini M Cell death and autophagy: cytokines, drugs, and nutritional factors Toxicology 2008 254 147 157 10.1016/j.tox.2008.07.048 18694801

[B59] Westfall MD Joyner AS Barbieri CE Livingstone M Pietenpol JA Ultraviolet radiation induces phosphorylation and ubiquitin-mediated degradation of DeltaNp63alpha Cell Cycle 2005 4 710 716 15846104 10.4161/cc.4.5.1685

[B60] Stampfer MR Garbe J Levine G Lichtsteiner S Vasserot AP Yaswen P Expression of the telomerase catalytic subunit, hTERT, induces resistance to transforming growth factor beta growth inhibition in p16INK4A(-) human mammary epithelial cells Proc Natl Acad Sci USA 2001 98 4498 4503 10.1073/pnas.071483998 11287649 PMC31863

[B61] Westfall MD Mays DJ Sniezek JC Pietenpol JA The Delta Np63 alpha phosphoprotein binds the p21 and 14-3-3 sigma promoters in vivo and has transcriptional repressor activity that is reduced by Hay-Wells syndrome-derived mutations Mol Cell Biol 2003 23 2264 2276 10.1128/MCB.23.7.2264-2276.2003 12640112 PMC150720

[B62] Mizushima N Yamamoto A Hatano M Kobayashi Y Kabeya Y Suzuki K Tokuhisa T Ohsumi Y Yoshimori T Dissection of autophagosome formation using Apg5-deficient mouse embryonic stem cells J Cell Biol 2001 152 657 668 10.1083/jcb.152.4.657 11266458 PMC2195787

[B63] Stewart ZA Leach SD Pietenpol JA p21(Waf1/Cip1) inhibition of cyclin E/Cdk2 activity prevents endoreduplication after mitotic spindle disruption Mol Cell Biol 1999 19 205 215 9858545 10.1128/mcb.19.1.205 PMC83879

[B64] Szak ST Mays D Pietenpol JA Kinetics of p53 binding to promoter sites in vivo Mol Cell Biol 2001 21 3375 3386 10.1128/MCB.21.10.3375-3386.2001 11313463 PMC100259

[B65] Rosenbluth JM Johnson K Tang L Triplett T Pietenpol JA Evaluation of p63 and p73 antibodies for cross-reactivity Cell Cycle 2009 8 3702 3706 10.4161/cc.8.22.10036 19855172

