# Supplementary figures and images for: ISG20L1 is a p53 family target gene that modulates genotoxic stress-induced autophagy
Source: Mol Cancer. 2010 Apr 29;9:95. doi: 10.1186/1476-4598-9-95 (PMC2873442; doi:10.1186/1476-4598-9-95)

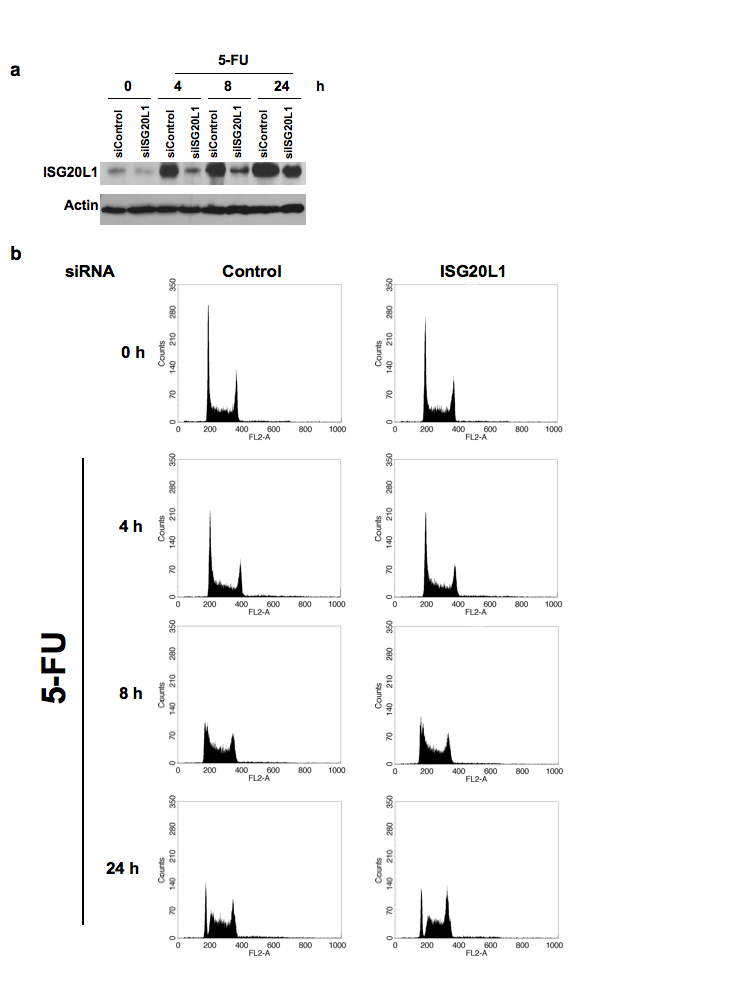

Supplement: Additional file 1 — Knockdown of ISG20L1 does not alter cell cycle. (a) U2OS cells were reverse transfected with nonsilencing control or siRNA targeting ISG20L1 and three days later treated or not with 5-FU over the indicated timecourse. Western analysis was performed to measure ISG20L1 and actin. (b) Flow cytometry was performed for each condition over the timecourse performed in (a) and a representative example of three independent experiments is shown. [file 1476-4598-9-95-S1.TIFF]

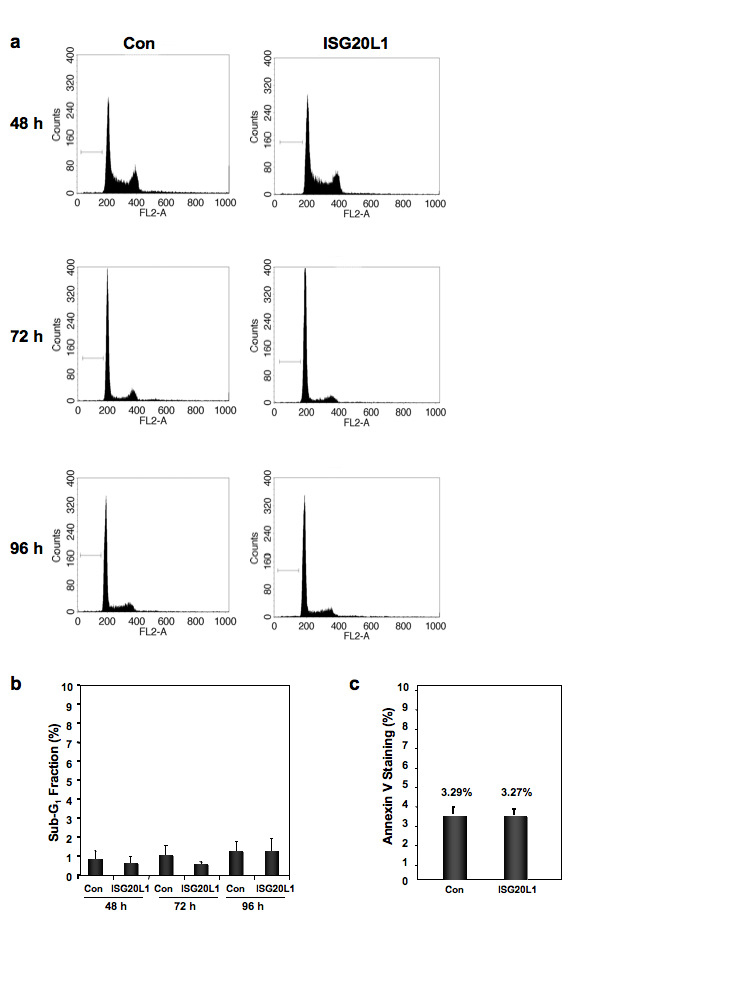

Supplement: Additional file 2 — The decrease in clonogenic survival after ectopic ISG20L1 expression is not accompanied by increased apoptosis. (a) Flow cytometry was performed over a timecourse with H1299 cells ectopically expressing either vector control or ISG20L1. A representative example of three separate experiments is shown. (b) The sub-G1percentage was analyzed for those samples described in part (a). (c) To further assess apoptosis, Annexin-V staining and flow cytometry were performed on H1299 cells 48 h after transfection with vector control or ISG20L1 and percent of cells stained for Annexin-V under both experimental conditions shown from three experiments. The error bars represent standard deviation. [file 1476-4598-9-95-S2.TIFF]
